# Protection of Blood Retinal Barrier and Systemic Vasculature by Insulin-Like Growth Factor Binding Protein-3

**DOI:** 10.1371/journal.pone.0039398

**Published:** 2012-07-06

**Authors:** Yagna P. R. Jarajapu, Jun Cai, Yuanqing Yan, Sergio Li Calzi, Jennifer L. Kielczewski, Ping Hu, Lynn C. Shaw, Sue M. Firth, Tailoi Chan-Ling, Michael E. Boulton, Robert C. Baxter, Maria B. Grant

**Affiliations:** 1 Department of Pharmacology and Therapeutics, College of Medicine, University of Florida, Gainesville, Florida, United States of America; 2 Department of Anatomy and Cell Biology, College of Medicine, University of Florida, Gainesville, Florida, United States of America; 3 Discipline of Anatomy, Bosch Institute, University of Sydney, Sydney, Australia; 4 Kolling Institute of Medical Research, University of Sydney, St. Leonards, New South Wales, Australia; University of Frankfurt - University Hospital Frankfurt, Germany

## Abstract

Previously, we showed that insulin growth factor (IGF)-1 binding protein-3 (IGFBP-3), independent of IGF-1, reduces pathological angiogenesis in a mouse model of the oxygen-induced retinopathy (OIR). The current study evaluates novel endothelium-dependent functions of IGFBP-3 including blood retinal barrier (BRB) integrity and vasorelaxation. To evaluate vascular barrier function, either plasmid expressing IGFBP-3 under the regulation of an endothelial-specific promoter or a control plasmid was injected into the vitreous humor of mouse pups (P1) and compared to the non-injected eyes of the same pups undergoing standard OIR protocol. Prior to sacrifice, the mice were given an injection of horseradish peroxidase (HRP). IGFBP-3 plasmid-injected eyes displayed near-normal vessel morphology and enhanced vascular barrier function. Further, in vitro IGFBP-3 protects retinal endothelial cells from VEGF-induced loss of junctional integrity by antagonizing the dissociation of the junctional complexes. To assess the vasodilatory effects of IGFBP-3, rat posterior cerebral arteries were examined *in vitro*. Intraluminal IGFBP-3 decreased both pressure- and serotonin-induced constrictions by stimulating nitric oxide (NO) release that were blocked by L-NAME or scavenger receptor-B1 neutralizing antibody (SRB1-Ab). Both wild-type and IGF-1-nonbinding mutant IGFBP-3 (IGFBP-3NB) stimulated eNOS activity/NO release to a similar extent in human microvascular endothelial cells (HMVECs). NO release was neither associated with an increase in intracellular calcium nor decreased by Ca^2+^/calmodulin-dependent protein kinase II (CamKII) blockade; however, dephosphorylation of eNOS-Thr^495^ was observed. Phosphatidylinositol 3-kinase (PI3K) activity and Akt-Ser^473^ phosphorylation were both increased by IGFBP-3 and selectively blocked by the SRB1-Ab or PI3K blocker LY294002. In conclusion, IGFBP-3 mediates protective effects on BRB integrity and mediates robust NO release to stimulate vasorelaxation via activation of SRB1. This response is IGF-1- and calcium-independent, but requires PI3K/Akt activation, suggesting that IGFBP-3 has novel protective effects on retinal and systemic vasculature and may be a therapeutic candidate for ocular complications such as diabetic retinopathy.

## Introduction

Hepatic insulin-like growth factors (IGF-1 and IGF-2) circulate almost entirely (>99%) bound to binding proteins (IGFBPs), of which there are six. IGFBP-3 is the most abundant binding protein and the major IGFBP species in the adult circulation [1]. IGFBP-3 binds 75 to 90% of circulating IGFs in a large ternary complex that consists of IGFBP-3, the acid-labile subunit (ALS) and IGFs [2]. ALS, produced by the liver, reduces the passage of IGF-1 to the extravascular compartment and stabilizes the IGF–IGFBP-3 complex, extending its half-life in serum [3]. Thus, the principal function of circulating IGFBP-3, in addition to the transport of IGFs, is the protection of the IGFs from rapid clearance and/or degradation [4]. At the cellular level, it has become clear that IGFBPs 1–6 have intrinsic biological activity (i.e., IGF/IGF-1R-independent actions) in addition to binding of IGFs, sequestering active hormones, and limiting IGF biological activity [5], [6]. These intrinsic cellular actions include proliferation, differentiation, migration, angiogenesis, and apoptosis in an IGF/IGF-1 receptor (IGF-1R)-independent manner [1], [7], [8].

By definition, a vasoprotective substance facilitates perfusion to ischemic areas, reduces endothelial apoptosis, recruits precursor cells to sites of injury, and prevents microvascular leakage. To date, IGFBP-3 has been shown to perform several of these functions, however, its effects on vascular permeability in the developing retina have not been studied and the mechanism for its vascular protective effect is largely unknown. Previously, in the oxygen-induced retinopathy (OIR) model, administration of IGFBP-3 resulted in reduced vaso-obliteration, that is protection of the developing vasculature from hyperoxia-induced regression, leading to a reduction in preretinal neovascularization.

IGFBP-3 expression has been shown to be increased in response to hypoxia, suggesting that it may represent part of the physiological response of a tissue to injury [9]–[11]. Granata et al [12] showed evidence for an IGF-1-dependent angiogenic response of IGFBP-3 and further proposed that the sphingosine kinase (SK)/sphingosine-1 phosphate (S1P) pathway is involved in this response.

Much like IGFBP-3, nitric oxide (NO) is considered a vasoprotective molecule at physiological concentrations and represents a multifunctional signaling molecule in the regulation of vascular tone and permeability under physiological conditions [13]. Physiological concentrations of NO protect the blood retinal barrier (BRB) from loss of integrity [14], whereas supraphysiological concentrations lead to breakdown of the BRB following injury [15]–[17]. Recently, we showed that IGFBP-3 can activate endothelial eNOS and stimulate NO generation by activation of the scavenger receptor–B1 (SRB1), suggesting that the vasoprotective effects of IGFBP-3 appear to be mediated in part by its ability to stimulate NO generation.

In this study, we tested whether IGFBP-3 can influence BRB function in developing mouse retina and in vitro. We also examined whether IGFBP-3 can modulate intraluminal pressure, a physiological stimulus that represents the basis of the pressure-dependent autoregulation of organ blood flow [18]. We delineated the specific signaling pathways that mediate IGFBP-3-dependent NO release. We showed that 1) IGFBP-3 stimulated eNOS activity and is associated with enhanced dephosphorylation of eNOS-Thr^495^; 2) NO release is IGF-1 independent, but not associated with an increase in intracellular calcium or decreased by blockade of Ca^2+^/calmodulin-dependent protein kinase II (CamKII); and 3) IGFBP-3 induced NO release was associated with an increase in phosphatidylinositol 3-kinase (PI3K) activity, Akt-Ser^473^ phosphorylation and selectively blocked by the SRB1-Ab or PI3K inhibitor LY294002. IGFBP-3 displays novel protective effects on retinal and systemic vascular beds.

## Methods

### Ethics Statement

Animal procedures were reviewed and approved by the Institutional Animal Care and Use Committee (IACUC) of the University of Florida (200902518). The investigation conforms to the *Guide for the Care and Use of Laboratory Animals* published by the U.S. National Institutes of Health (NIH Publication No. 85–23, revised 1996). All animals were treated in accordance with the Guiding Principles in the Care and Use of Animals (NIH) and the ARVO Statement for the Use of Animals in Ophthalmic and Vision Research.

### OIR Model and Intravascular Perfusion of Horse Radish Peroxidase (HRP)

Pregnant C57BL/6 mice were purchased from The Jackson Laboratory (Bar Harbor, ME). A total of 20 mouse pups were used as previously described [19].

The IGFBP-3 plasmid, under the control of a proliferating endothelial cell-specific promoter, was injected into the eye (0.5 µl of 1 µg/1 µl solution) on postnatal day 1. The proliferating endothelial promoters were composed of a 4×(1297) 46-mer multimerized endothelin enhancer (ET_e_) upstream of a human Cdc6 promoter (Cdc6_p_) [20]. Then on post natal day 7, mice were placed with their nursing dams in a 75% oxygen atmosphere for 5 days. The barrier properties of retinal vessels in the mouse OIR model were determined by intravascular injection of HRP (Roche Diagnostics Corporation Indianapolis, IN) on postnatal day 17. The pups were given intra-vascular injections of HRP (40 mg/100 g body weight) dissolved in 0.3 ml Hartman’s solution into the retro-bulbar sinus, 30 minutes before sacrifice. Pups were placed on a weigh tray that was located over crushed ice to maintain the pups motionless during the procedure. This represented an alternative to anesthesia. The animal was sacrificed using isoflurane followed by cervical dislocation. The anterior segment and vitreous humor were quickly removed into ice-cold phosphate-buffered saline (PBS), and the eyecups immersed and fixed in ice-cold 4% (w/v) paraformaldehyde for 1 hour following Chan-Ling [21]. The HRP reaction product was visualized using nickel enhancement in the presence of diaminobenzidine (DAB). Retinas were washed in 0.1M PBS at 7.4, followed by another wash in nickel Tris-buffered saline (TBS) (0.04%) at pH 7.4 for 10 minutes. The peroxidase was visualized by applying 0.05% DAB and hydrogen peroxide (0.02%) in nickel TBS following Chan-Ling et al [22]. The duration of this incubation was determined by observation of the specimen under a dissecting microscope and stopped when optimal contrast between the label and the background was achieved. To avoid loss of HRP from within the vessel lumen, the retinas were fixed and reacted with peroxidase as an eyecup prior to placement of the radial incisions to permit flattening of the retina. The retinal whole mounts were then mounted in PBS/glycerol for observation using a Zeiss Axioplan 2 deconvolution microscope (model HBO 100) and Axiocam HRm camera. For each retina, images labeled with HRP were obtained at 20 times magnification (field size  = 400×533 um). Four fields of views of the superficial and deep vascular plexus were captured with the 20X objective and analyzed using LMS 510 software to provide a quantitative index of HRP retention, where an index of 1, is assumed for age- matched controls. The HRP average intensity was determined within the vessel lumen and in the immediate adjacent parenchyma, where luminal values acted as the denominator. For each field of view, the “Average Intensity” was determined for five regions of interest (ROI) using the LMS 510 software (Carl Zeiss, Jena Germany).

**Figure 1:**
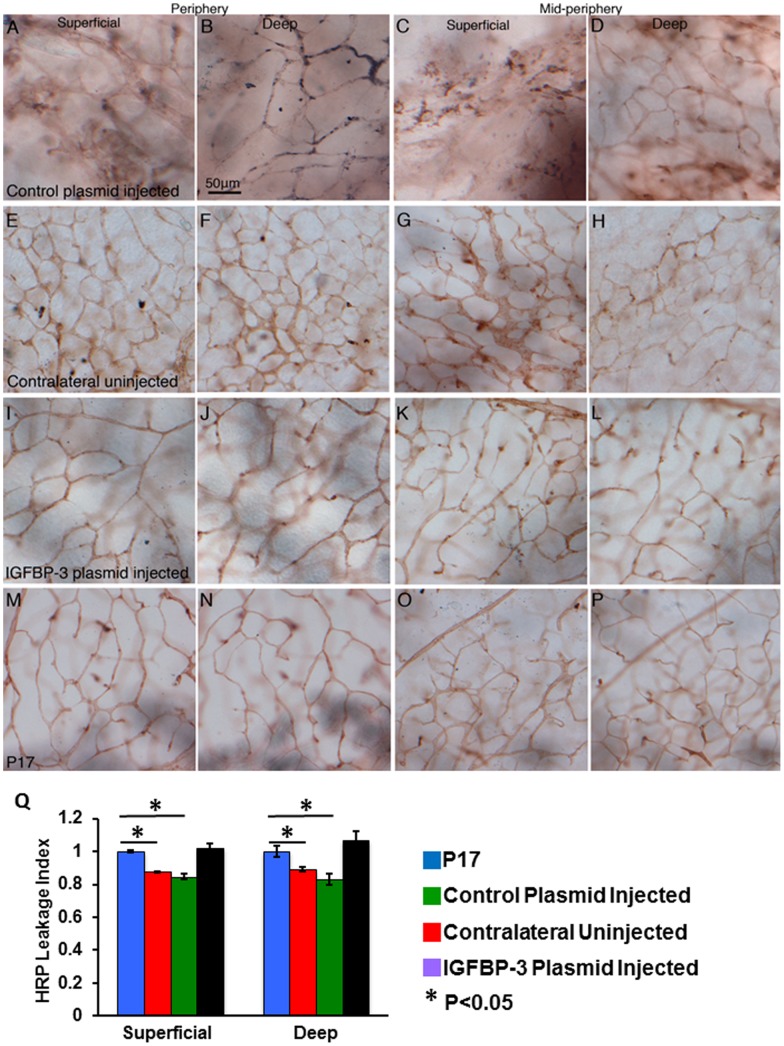
IGFBP-3 decreases vascular leakiness in the OIR model at P17. **A–P** Retinal whole mounts following intravascular perfusion of HRP in OIR control plasmid injected eyes (A–D), contralateral uninjected eyes (**E–H**), OIR IGFBP-3 plasmid injected eyes (**I–H**) and control animals (**M–P**) in the peripheral and mid-peripheral regions. In the contralateral uninjected eye, HRP had leaked from within the vessel lumen and was evident in the tissue parenchyma as a brown background staining. The outlines of the vessels were not sharp with diffuse HRP reaction product (brown color) in the parenchyma. The control plasmid injected eyes had more leaking. In contrast, both the IGFBP-3-injected eyes and P17 control eyes showed high levels of contrast between the vessel lumen and the tissue parenchyma, illustrating that the HRP reaction product was well retained within the vessel lumen and indicating an intact BRB since HRP has a similar molecular weight to albumin. **Q** The HRP average intensity was determined within the vessel lumen and in the immediate adjacent parenchyma where luminal values acted as the denominator. The superficial and deep vascular plexii were captured and analyzed using LMS 510 software to provide a relative quantitative index of HRP retention, where an index of 1, is assumed for age-matched controls. During the hypoxic phase of OIR, in the neovasculature of the contralateral uninjected eyes had a HRP leakage index of 0.875±0.006 in the superficial plexus and 0.890±0.014 in the deep plexus (P<0.05, Kruskal-Wallis test). The HRP leakage index in control plasmid injected retinas were 0.847±0.016 in superficial plexus and 0.833+0.033 in deep plexus (P<0.05, Kruskal-Wallis test). In contrast, IGFBP-3 plasmid injected eyes had a HRP leakage index of 1.023±0.025 in the superficial plexus and 1.070±0.051 in the deep layer compared with an index of 1 for the age-matched control eyes (not statistically significant) indicative of the enhanced barrier function of the neovascularization of the OIR model after IGFBP-3 injection. The barrier properties of the vessels in IGFBP-3 injected eyes was found to be significantly (P<0.05) higher than contralateral uninjected eyes or plasmid-injected eyes, similar to that observed in healthy P17 control eyes in both superficial and deep vascular plexuses. Calibration in B is applied through A to P.

### Ex vivo Whole Vessel Studies

To examine the direct effect of IGFBP-3 on vasculature, we examined another vascular bed that demonstrates robust barrier characteristics, the cerebral arteries. To study cerebral vessels, we used male Sprague-Dawley (SD) rats (250–300 g). The rats were asphyxiated with carbon dioxide and then decapitated and their brains were removed and placed in an ice-cold oxygenated physiological saline solution (PSS). Posterior cerebral arteries (PCAs) were isolated and cannulated with glass pipettes mounted in an arteriograph (Living Systems Inc., Burlington, VA) and placed on the stage of an inverted microscope for the diameter measurement as described earlier [23]. For these studies, IGFBP-3 and the non-IGF-binding mutant were expressed in 911 human retinoblastoma cells and purified as previously described [24]. IGFBP-3 or the non-IGF-binding mutant was used at concentration of 100 ng/ml. IGFBP-3, its vehicle (10 mM acetic acid) or blockers (either L-NAME or SRB1 neutralizing antibody (SRB1-Ab)) were applied intraluminally to the posterior cerebral arteries. Arterial segments were mounted in the arteriograph with the cannulae filled with either PSS or 10 mM acetic acid or IGFBP-3. To examine the effects of L-NAME or SRB1-neutralizing antibody (SRB1-Ab), arterial segments were mounted with the cannulae filled with blockers and after an hour, the solution in the cannulae was replaced with PSS containing the blocker and IGFBP-3. After an equilibration period of approximately 30 minutes, arteries were slowly pressurized to 70 mmHg. To evaluate constriction to different pressures, intraluminal pressure was increased slowly from 10 to 100 mmHg in increments of 30. At each pressure step, arteries were allowed to equilibrate for a minimum of 10 minutes or until they showed a stable diameter. Concentration response curves (CRCs) to the contractile agonist, serotonin, were generated in arteries pressurized at 10 mmHg, during which the activation of myogenic mechanisms were minimal. All experiments ended with the arteries exposed to calcium-free PSS to determine the passive diameter at different intraluminal pressures. Constriction in response to pressure, myogenic tone, was calculated according to the following equation:

Myogenic tone  =  (Dp-Da)/Dp * 100 (1)

where Da is the internal diameter of the arterial segment with active myogenic tone in the presence of PSS at a particular intraluminal pressure and Dp is the passive diameter.

### Immunostaining of VE-cadherin and Claudin-5 in Retinal Endothelial Cells

To better characterize the impact of IGFBP-3 on the BRB, we performed immunohistochemistry of the adherence junction protein, VE- cadherin and of the tight junction protein, claudin -5 using an in vitro system that recapitulates aspects of the BRB. Bovine retinal microvascular endothelial cells were isolated from freshly obtained retinas and cultured in MCDB131 medium with growth supplement (Invitrogen, CA) as described previously [25]. To carry out immunocytochemistry, cells were cultured on glass bottom microwell dishes (MatTek Corp., MA) coated with attachment factors. At confluence cells were exposed to either IGFBP-3, VEGF or both IGFBP- 3 and VEGF for up to 12 hrs and then fixed with 4% paraformaldehyde plus 4% sucrose in PBS and permeabilized with 0.1% Triton X-100. Following 30 min exposure to 5% BSA in PBS at room temperature, cells were incubated with primary antibodies for VE-cadherin (goat polyclonal, Santa Cruz, CA) and claudin-5 (rabbit polyclonal, Abcam, MA) at 1∶1000 in PBS with 5% BSA at 4°C overnight. Donkey anti-goat IgG secondary antibodies for VE-cadherin (Alexa Fluor 549-labeled) and claudin-5 (Alexa Fluor 488-labeled) (Invitrogen, CA) at 1∶1000 in 5% BSA in PBS at room temperature for 1 hour in the dark. Negative control treatments were carried out by excluding primary antibodies. Digital fluorescence microscopic evaluation of the immunostaining was carried out by using spinning disk confocal microscope (Olympus IX81-DSU).

**Figure 2:**
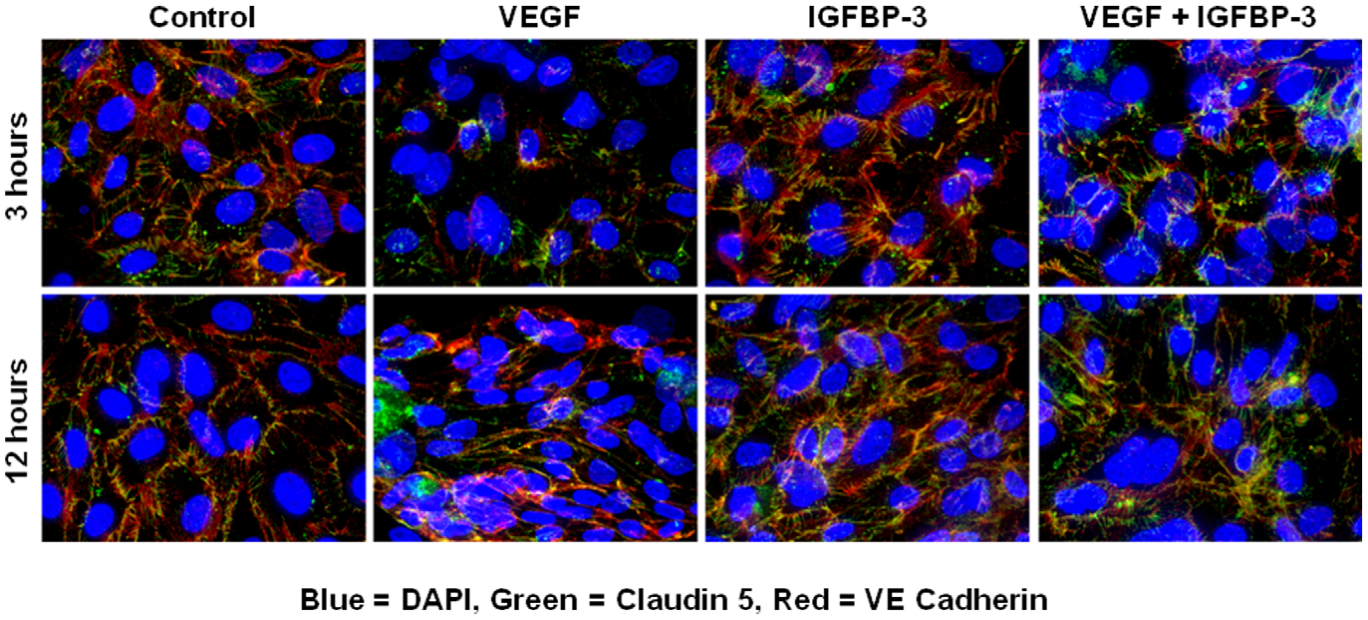
IGFBP-3 inhibits VEGF-mediated disruption of junctional protein complex. Immunocytochemistry of claudin and VE-cadherin in monolayers of bovine microvascular endothelial cells. Treatment with VEGF caused dissociation of junctional proteins at 3 hours and the effect was lower at 24 hours following treatment. IGFBP-3 has no effect on the junctional protein complexes however it antagonized the dissociation of the junctional complexes produced by VEGF. Shown were representative confocal microscopic images of claudin and VE-cadherin immunochemistry with DAPI as the nuclear stain obtained from three independent experiments.

### Fluorescence Imaging of NO

To evaluate NO generation in intact arteries, arterial segments were loaded with DAF-FM diacetate (Invitrogen, Carlsbad, CA, USA), an NO-sensitive fluorescent dye, intraluminally with the cannula filled with PSS containing 10 µM DAF-FM for approximately 30 min. Then, the solution in the cannula was replaced with PSS containing IGFBP-3. The arteriograph was placed on the microscope for fluorescence microscopy, and the temperature of PSS slowly increased to 37°C as described above. Arterial segments were slowly pressurized to 70 mmHg. Fluorescence images were obtained when arteries showed a stable diameter using a computer controlled monochromatic excitation light source (TILL Polychrome II, TILL-photonics, Martinsried, Germany) and a cooled CCD camera with exposure control (SensiCam, TILL-Photonics). Images were acquired by Till-Vision software (TILL-photonics) using a10X-fluor objective at excitation and emission wavelengths of 488 and 535 nm, respectively. Offline analysis of images was carried out using Till-Vision and Microsoft Excel.

**Figure 3:**
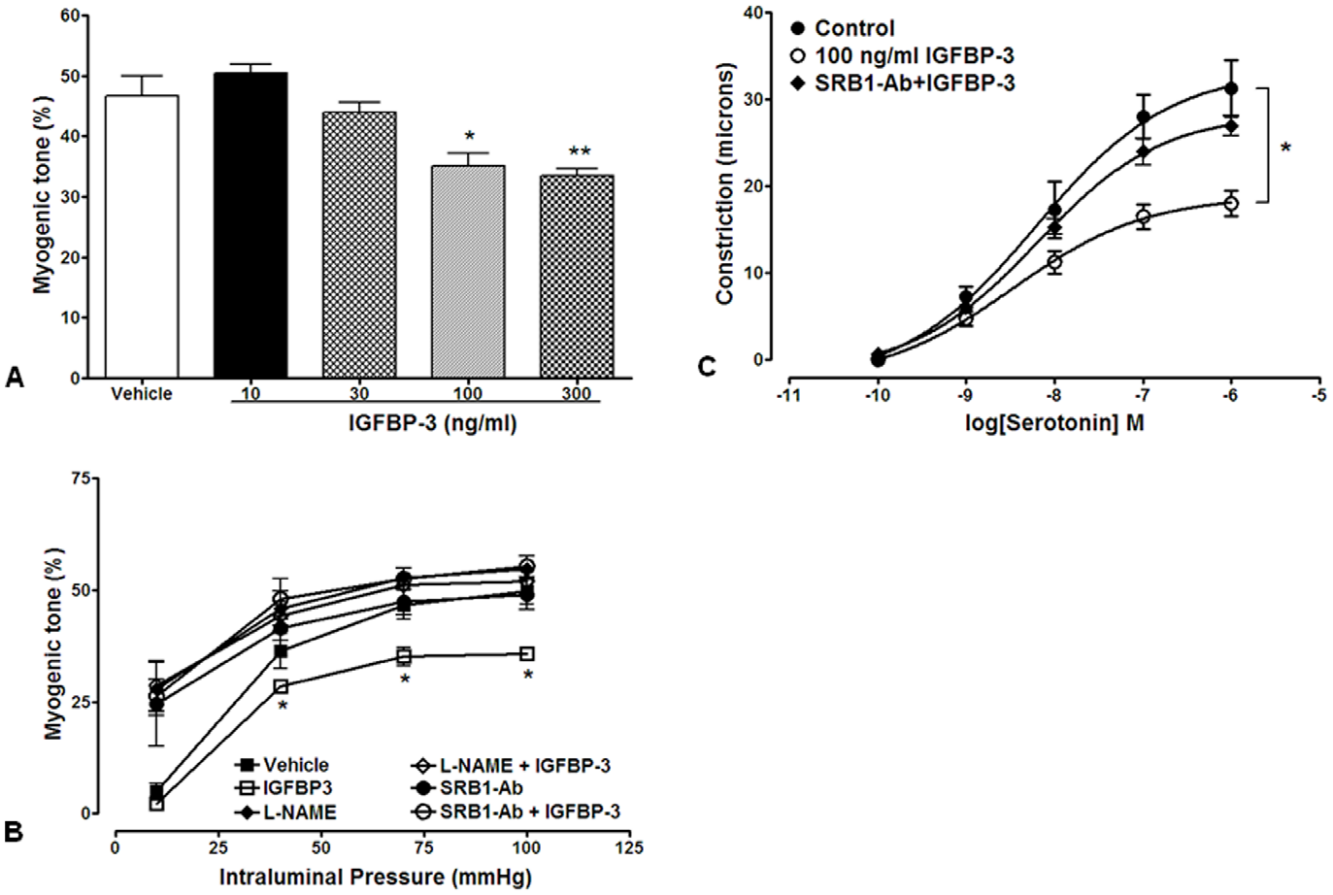
IGFBP-3 decreases vascular tone by activating scavenger receptor-B1. **A**. Concentration-dependent effects of intraluminally applied IGFBP-3 on myogenic tone in rat PCAs pressurized at 70 mmHg. A significant decrease in myogenic tone was observed at 100 (*P<0.02, n = 5) and 300 ng/ml (**P<0.01, n = 5) IGFBP-3. **B**. Effects of intraluminal IGFBP-3 on myogenic tone developed in response to intraluminal pressures ranging from 10 to 100 mmHg (n = 6). Decrease in myogenic tone by 100 ng/ml IGFBP-3 was significant at pressures 40 (P<0.05), 70 (P<0.03) and 100 (P<0.01) mmHg compared to control (vehicle intraluminally applied). In the presence of intraluminal L-NAME (300 µM) or scavenger receptor-B1 ligand blocking antibody (SRB1-Ab) (1∶100) myogenic tone was not affected by IGFBP-3. **C**. Effects of intraluminal IGFBP-3 on serotonin-induced constriction in PCAs pressurized at 10 mmHg (n = 5). Concentration-response curves to serotonin were significantly lower in the presence of 100 ng/ml IGFBP-3 compared to those from control (*P<0.02 two-way ANOVA). In the presence of SRB1-Ab (1∶100) effects of IGFBP-3 were not observed.

### Fluorescence Microscopy in Cultured Endothelial Cells

To better understand the effect of IGFBP-3 on human cells, we examined human microvascular endothelial cells (HMVECs) in culture. HMVECs were obtained from Lonza (Walkersville, MD) and maintained as per the supplier’s instructions. For fluorescence microscopy, semi-confluent cells were trypsinized and replated in glass bottom microwell dishes (MatTek Corp., MA). Following an overnight incubation with serum-free medium, HMVECs were loaded with 10 µM 4-amino-5-methylamino-2′,7′-difluororescein diacetate (DAF-FM) for 30–45 minutes in Dulbecco’s (DPBS) containing calcium and magnesium (Mediatech, Inc., Manassas, VA) supplemented with glucose (1 mg/ml) and L-arginine (1 mM). The DAF-FM-loaded cells were placed on the stage of the Axiovert inverted microscope with a 20X fluor objective (Zeiss) for fluorescence imaging. Images were obtained and analyzed using Till-Vision software as described above to evaluate the effects of IGFBP-3 or 4α-phorbol 12,13-didecanoate (4αPDD) on NO generation. 4α-PDD is a robust and reliable tool to study nonselective cation channels, transient receptor potential vanilloid (TRPV) type channels, and to probe functional effects of the activation of this channel. Cells were treated with these agents 15 minutes after cells were loaded with DAF-FM and further incubated for 30 minutes. Some dishes were incubated with SRB1-Ab or L-NAME for 30 minutes before loading cells with DAF-FM. Changes in DAF fluorescence with different treatments were expressed as the percent change with respect to cells that were used as either time or vehicle control i.e. cells that received no treatments, but were loaded with DAF-FM.

### Fura-2 imaging in Cultured Endothelial Cells

To examine the intracellular Ca^2+^ levels, cells were plated in glass bottom dishes as described above and loaded with 5 µM fura-2 AM (Invitrogen) in DMSO with an equal volume of 10% w/v pluronic F-127 (Sigma) for 30 minutes. Fura-2 ratiometry was carried out using the TILL Polychrome at excitation wavelengths of 340 and 380 nm and an emission wavelengths of at 510 nm. A 340/380 ratio image was generated following background subtraction using Till-Vision software.

### Immunohistochemistry

Rat PCAs were cannulated, pressurized and fixed with intra- and abluminal 4% formaldehyde in PBS for 1 hour at room temperature, and all subsequent treatments were administered at room temperature. Arterial segments were removed from the cannulae, placed in a 96- well plate, and permeabilized with 2% Triton X-100 for 15 minutes. Following permeabilization, arterial segments were then washed with PBS and blocked with 2% bovine serum albumin (BSA) in PBS for 1 hour. The segments were washed with PBS and incubated with primary antibodies against SRB1 (rabbit polyclonal, NOVUS Biologicals, LLC., Littleton, CO) and eNOS (mouse monoclonal, Abcam Inc., Cambridge, MA) in 1% goat serum in PBS for 30 minutes followed by washing with PBS. Arteries were then incubated with secondary antibodies in PBS containing 0.1% BSA for 60 minutes followed by washing with PBS. Arterial segments were mounted with Vectashield ® mounting medium containing 4′,6-diamino-2-phenylindole (DAPI) for nuclear DNA staining (Vector Laboratories, Inc., Burlingame, CA) on a glass slide with its tubular structure intact. Digital fluorescent images were acquired using spinning disk confocal microscope (Olympus), and the images were processed offline using ImageJ software (ImageJ 1.37n; Wayne Rasband, NIH).

### eNOS Activity Assay

To establish whether IGFBP-3 has a similar effect on macrovascular endothelial cells, we examined eNOS activity in HMVECs. Activation of eNOS by IGFBP-3 was evaluated by measuring L-citrulline synthesis in HMVECs using radioactive L-arginine as substrate. Briefly, the cell suspension was incubated with L-[^14^C] arginine (American Radiolabeled Chemicals Inc.) at 37°C with constant agitation in the presence or absence of 500 µM L-NAME, a NOS inhibitor. Following incubation, cells were lysed by sonication for 10 seconds and the sample suspension was run through 1-mL columns of Dowex AG50WX-8 (Na^+^ form) (Sigma). Radioactivity corresponding to [^14^C]citrulline within the eluate was quantified by liquid scintillation counting. Enzyme activity was expressed as the radioactivity contained that was inhibited by L-NAME/mg of cell protein. To evaluate the effects of SRB1-Ab on IGFBP-3-stimulated eNOS activity, cell suspensions were incubated with blocker for 30 minutes before the addition of IGFBP-3.

### Western Blotting

Effects of IGFBP-3 on the phosphorylation of eNOS and Akt were evaluated by western blotting. HMVECs were cultured to semiconfluence as described above and were serum-starved overnight prior to the treatment with IGFBP-3. Pharmacological inhibitors or the vehicle were added to the cells 30 min before the treatment with IGFBP-3. At the end of the treatments, dishes were kept ice-cold, cells were lysed with RIPA buffer (Pierce, Rockford, IL) and protein was extracted. 50 micrograms of protein was loaded on to 10% polyacrylamide precast gels (Bio-Rad, Hercules, CA) and resolved proteins were transferred on to nitrocellulose membranes (Bio-Rad) using standard western blotting protocols. Total and phosphorylated eNOS and Akt proteins were immunoblotted using the following primary and secondary antibodies from Cell Signaling Technology, Inc. (Danvers, MA) - Akt (9272), and phospho-Thr^308^ Akt (5106) or from Santa Cruz Biotechnology, Inc. (Santa Cruz, CA): - phospho-Ser^473^ Akt (SC-7958-R) β-actin (sc-47778), goat antimouse IgG-HRP (sc-2005) and goat antirabbit IgG-HRP (sc-2370). Equal protein loading was ensured by probing for β-actin.

**Figure 4:**
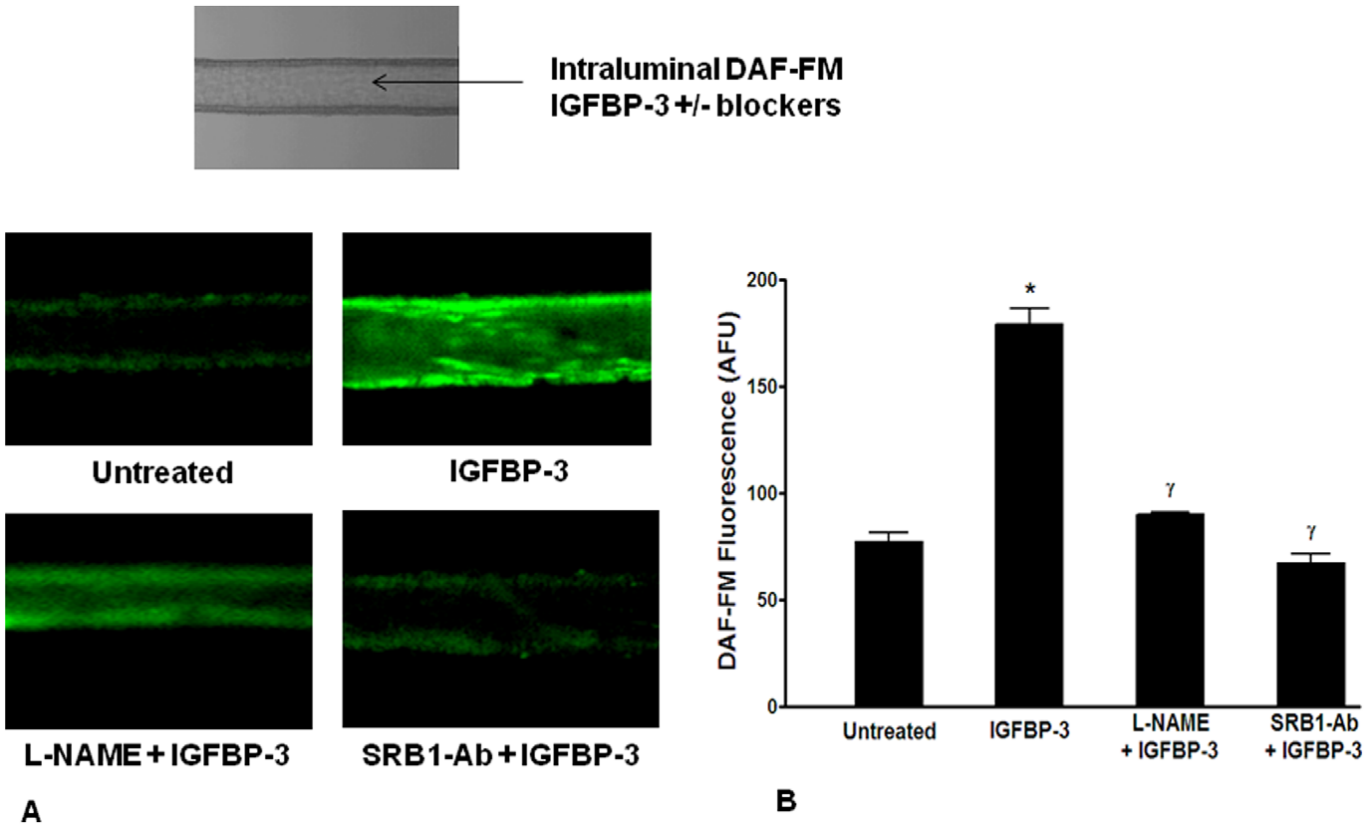
IGFBP-3 stimulates NO release in intact arteries by activating scavenger receptor-B1. **A**. Intraluminal IGFBP-3 increased NO generation, determined by DAF-FM fluorescence, in rat PCAs pressurized at 70 mmHg: i) bright field image of pressure-mounted artery; ii) DAF-FM fluorescence image of the arterial segment with vehicle intraluminally applied; iii) image of an artery treated with IGFBP-3, iv) image of an artery treated with L-NAME and IGFBP-3; and v) image of an artery treated with SRB1-Ab and IGFBP-3. Shown in the left was color scale for DAF-FM fluorescence. **B**. Quantification of the effect of IGFBP-3 in the presence or the absence of different blockers on myogenic tone and NO generation. DAF-FM fluorescence, expressed as arbitrary fluorescence units (AFU) was significantly increased (*P<0.02, n = 5) by IGFBP-3 compared to vehicle-control. Effects of IGFBP-3 were inhibited by 300 µM L-NAME (γ P<0.03, n = 4) or 1∶100 SRB1-Ab (Ψ P<0.02, n = 4).

**Figure 5:**
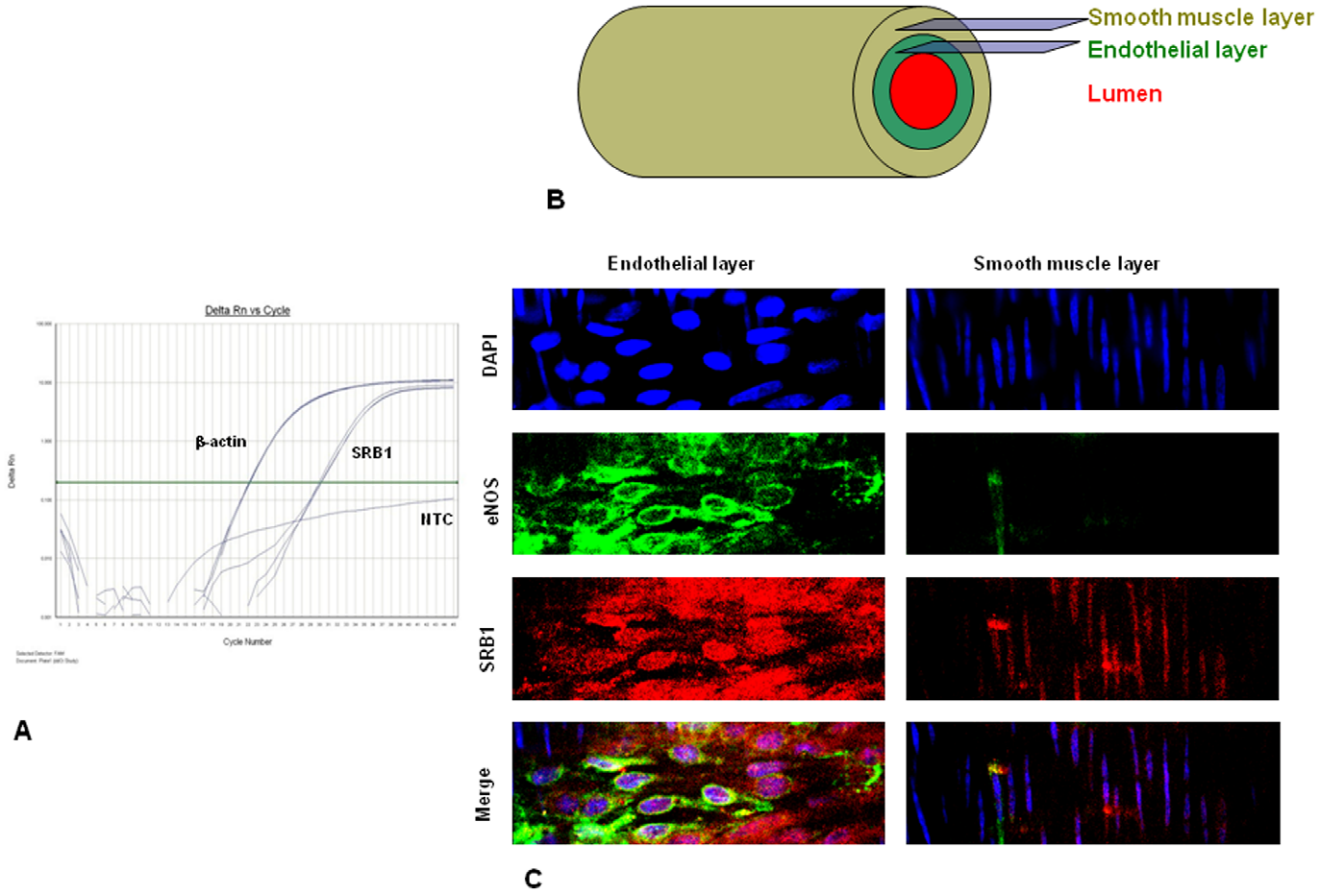
Scavenger receptor-B1 is expressed in rat cerebral arteries. **A**. A representative real-time PCR trace showing the detection of mRNA of rat scavenger receptor-B1 (SRB1) (CT: 29.0±0.2) in posterior cerebral arteries (PCAs) relative to that of β-actin (CT: 22.9±0.2, n = 3). NTC - no template control. **B**. schematic representation of the smooth muscle and endothelial layers in the arterial wall that were imaged for immunohistochemical localization of SRB1 and eNOS. **C**. Immunohistochemical localization of eNOS and SRB1 in rat PCAs. Left panel – endothelial layer; right panel – smooth muscle layer; Blue – DAPI, nuclear stain; green – eNOS; red – SRB1 and merge – composite image of three colors. SRB1 appears to be mostly localized in the endothelial layer, partly co-localized with eNOS, and nuclear localization is evident in endothelial cells and partly in smooth muscle cells.

### Real-time PCR

Expression of SRB-1 in rat PCAs was evaluated by real-time PCR. Rat PCAs were isolated and cleaned of luminal blood and total mRNA was isolated using an RNA Mini Kit (Bio-Rad, Hercules, CA). Arteries from 3 three rats were pooled per sample, and three samples were used for real-time PCR. The mRNA was transcribed using an iScript cDNA Synthesis Kit (Bio-Rad, Hercules, CA), and real-time PCR was performed using the ABI Master Mix (Applied Biosystems, Foster City, CA). Primers for rat SRB1 (Rn00580588_m1) and rat β-actin (Rn00667869_m1) were purchased from Applied Biosystems. Real-time PCR was performed in triplicate on a 7500 Fast PCR machine (Applied Biosystems) for 40 cycles.

Expression of the recently discovered death receptor for IGFBP-3 was evaluated in HMVECs using the primers (forward 5′-ATGGGACAGTCACAGGGAAG-3′; reverse 5′-AAGGCCACAGAAGAGAAGCA-3′) reported by Ingerman et al [26]. The following primers were used for β-actin: forward 5′-ATC AAG ATC ATT GCT CCT CCT GAG-3′; reverse 5′-AGC GAG GCC AGG ATG GA-3′. Total mRNA was isolated from endothelial cells and cDNA was obtained by reverse transcription as described above and real-time PCR was carried out using SYBR-green PCR master mix (Applied Biosystems). Expression of human SRB1 was evaluated by using gene expression assay Hs00969818_m1 relative to β-actin, Hs99999903_m1 (Applied Biosystems).

### Phosphatidyl Inositol-3 Kinase (PI3K) Activity Assay

Phosphatidyl inositol-3 kinase (PI3K) activity assay was performed by enzyme-linked immunosorbent assay (ELISA) K-1000s-PI3-kinase activity (ELISA:Pico, Echelon Biosciences Inc., Salt Lake City, UT,) as per the manufacturer’s instructions.

### Data Analysis and Statistics

Results are expressed as the mean±SEM; ‘n’ indicates the number of independent experiments, which equals the number of animals used, where applicable. Results were compared by Students ‘t’-test or two-way ANOVA using GraphPad Prism software (San Diego, CA). Non-parametric analysis, the Kruskal-Wallis test, was used where appropriate. ‘P’ value of less than 0.05 was considered statistically significant.

**Figure 6:**
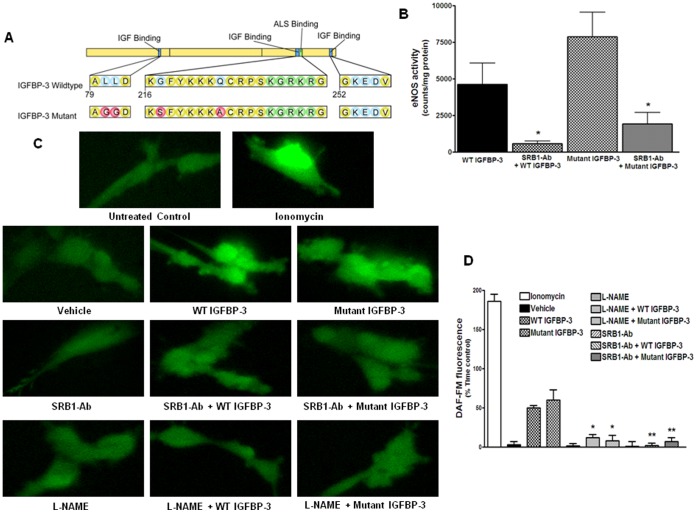
Effects of wild-type and IGF1-non-binding mutant IGFBP-3 are similar in stimulating NO generation. **A**. Schematic of the amino acid sequence in wild-type (WT) IGFBP-3 and mutations that prevent its binding to IGF-1. Changes in the amino acids of the mutant IGFBP-3 were indicated in red. **B**. Activity of eNOS expressed as the counts inhibited by L-NAME/mg protein in HMVECs. Both WT and mutant IGFBP-3 stimulated eNOS activity to a similar extent and their effects were attenuated by scavenger receptor-B1 neutralizing antibody (SRB1-Ab) (*P<0.005, n = 3). **C**. Determination of NO generation in response to WT or mutant IGFBP-3 by DAF-FM fluorescence microscopy in HMVECs. Shown in the left was color scale for DAF-FM fluorescence. Up to 20–40 cells were imaged per treatment in three independent experiments. Ionomycin is a positive control that produced robust increase in NO generation. **D**. Quantification of the effects of WT or mutant IGFBP-3 in the presence or absence of different blockers on NO generation. Both WT and mutant IGFBP-3 stimulated NO generation to similar extents. Pretreatment with 300 nM L-NAME significantly decreased the effects of WT or mutant IGFBP-3 (*P<0.002). Similarly, pretreatment with SRB1-Ab decreased the effects of WT or mutant IGFBP-3 (**P<0.001).

**Figure 7:**
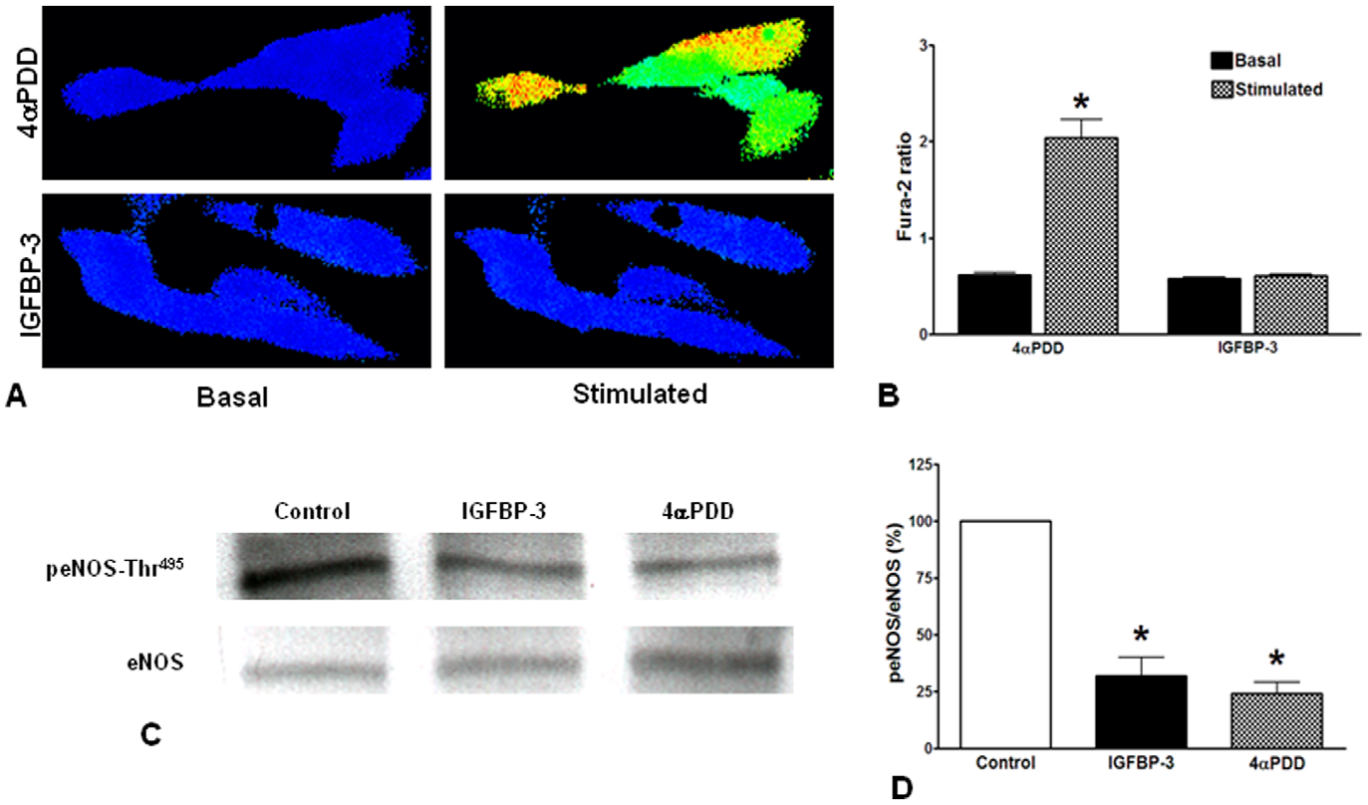
IGFBP-3 causes eNOS activation by dephosphorylation of Thr^495^ independent of intracellular calcium. **A**. Effects of IGFBP-3 or 4αPDD on intracellular calcium levels in HMVECs determined by Fura-2. Shown were the representative images of Fura-2 ratio from four independent experiments. Shown in the left was color scale for Fura-2 ratio images. **B**. Quantification of the effects of 4αPDD and IGFBP-3 on intracellular calcium: 4αPDD (10 µM) produced a robust increase in intracellular calcium whereas no change was observed in response to 100 ng/ml IGFBP-3. **C**. Western blotting of eNOS and eNOS phosphorylation at the Thr^495^ residue. **D**. Densitometry of three independent experiments. Treatment with either IGFBP-3 or 4αPDD decreased the phosphorylation of Thr^495^ residue significantly (*P<0.01) compared to control. Values are expressed as percentage of phospho-eNOS to eNOS ratio.

**Figure 8:**
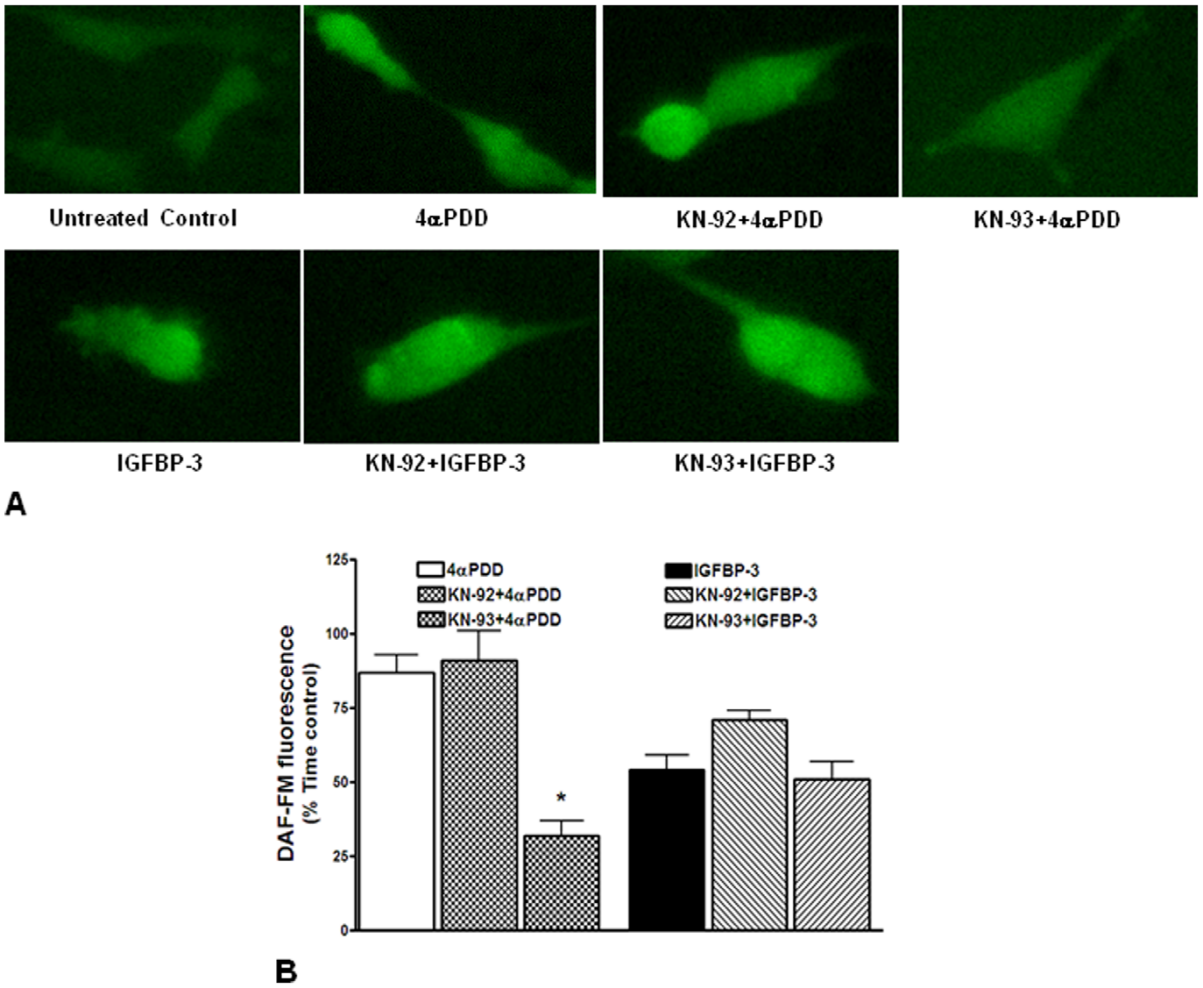
NO release by IGFBP-3 is independent of CamKII activation. **A**. Determination of NO generation in response to 4αPDD or IGFBP-3 by DAF-FM fluorescence microscopy in HMVECs. Shown were color scale for DAF-FM fluorescence and representative images of HMVECs with different treatments. UP to 40 cells were imaged per treatment in three independent experiments. **B**. Quantification of DAF-FM fluorescence expressed as percent of time control illustrating the effects KN93, CamKII blocker, and its negative control KN92 on NO generation by 4αPDD or IGFBP-3. NO generation by 4αPDD was significantly attenuated by KN93 (*P<0.02), but not by KN92, whereas NO generation by IGFBP-3 was not affected by either KN93 or KN92.

## Results

### IGFBP-3 Enhances Blood-retinal Barrier Integrity in the Neovasculature of OIR Mice

To determine whether IGFBP-3 modulates BRB integrity, we injected IGFBP-3 expressing or control plasmid into the vitreous humor of mouse pups (post natal day 1, P1) following the standard OIR protocol (Figure 1A–P). Mice were withdrawn from high oxygen at P12 and sacrificed at P17 during the hypoxic vasoproliferative stage of OIR. As seen in control eyes, vaso-proliferation is characterized by capillary networks showing variation in vessel caliber and irregular branching patterns (Figure 1E–F). Vessels with lumen diameters up to 10–20 µm were evident in these eyes. The density of HRP injected within the vasculature showed a great variation within different segments of the vascular tree, indicative of varying barrier properties along the vessel length. The intensity of the HRP reaction product within the vessel lumen was significantly reduced in the non-injected or control plasmid-injected eyes, indicative of leakiness from the vessel lumen. Furthermore, the parenchyma of the control plasmid- treated eyes had a high level of background staining as much of the HRP had leaked from within the vessel lumen (Figure 1A–D). The leakiness of the retinal vessels was quantified by assessing HRP densities within vessel lumens and in the adjacent tissue parenchyma using the “average intensity” function of the LSM510 software. This was determined in 4 fields of view and expressed as a ratio where the value for a P17 age-matched healthy mouse (no OIR) was used as the denominator, resulting in the age-matched control mouse having a HRP leakage index of 1. During the hypoxic phase of OIR, the neovasculature of the contralateral non-injected eyes had an HRP leakage index of 0.875±0.006 in the superficial plexus and 0.890±0.014 in the deep plexus (P<0.05, Kruskal-Wallis test). The HRP leakage index in plasmid injected retinas were 0.847±0.016 in superficial plexus and 0.833+0.033 in deep plexus (P<0.05, Kruskal-Wallis test). In contrast, IGFBP-3 injected eyes had a HRP leakage index of 1.023±0.025 in the superficial plexus compared to 1.070±0.051 in the deep plexus with an index of 1 for the age-matched control eyes (not statistically significant) indicative of the enhanced barrier function of the neovascularization of the OIR model with IGFBP-3 plasmid injection (Figure 1Q). This enhancement of the BRB by IGFBP-3 plasmid injection is accompanied by significant normalization of the vessel morphology (Figure 1 I-L). The capillary tree had near normal vessel caliber and meshwork morphology. Furthermore, the vessel lumens were characterized by retention of HRP reaction product, resulting in a very light parenchyma without obvious HRP leakage. When the IGFBP-3 plasmid- injected pups undergoing the OIR model were compared to normal healthy P17 pups reared in normal oxygen from birth, the P17 mice had similar retinal vessel morphology and barrier properties as the IGFBP-3 injected eyes of the OIR model (Figure 1 M-P).

### IGFBP-3 Protects Retinal Endothelial Cells from VEGF-induced Loss of Junctional Integrity

In order to better understand the protective function of IGFBP-3 on retinal vascular permeability, we have evaluated the effect of IGFBP-3 on VEGF-induced disruption of junctional complexes by performing immunohistochemistry of claudin and vascular endothelial cadherin (VE-cadherin) in monolayers of bovine retinal microvascular endothelial cells. As shown in Figure 2, VEGF (100 ng/ml) treatment caused dissociation of claudin and VE-cadherin by 3 hrs and this dissociation tended to recover by 12 hrs. IGFBP-3 (100 ng/ml) alone did not have any effect on the integrity of junctional complexes at 3 and 12 hrs of treatment. However, in the presence of IGFBP-3, VEGF-induced dissociation of claudin and VE-cadherin was completely blocked (Figure 2). These results suggest that the protection from vascular leakage by IGFBP-3 observed in the in vivo experiments could be, in part, due to rescuing the integrity of junctional complexes from the deleterious effects of VEGF. Increased VEGF expression in the neovascularization phase of the OIR model has been well established [27].

**Figure 9:**
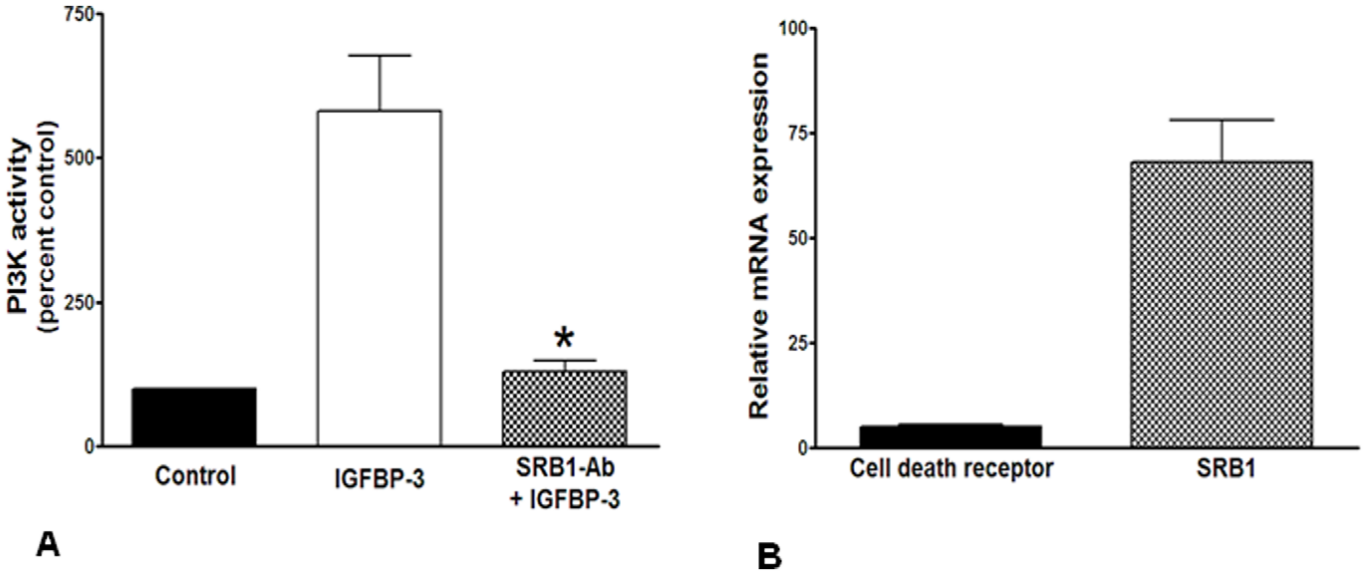
IGFBP-3 activates PI3kinase via scavenger receptor-B1. **A**. PI3K activity was evaluated in HMVECs and expressed as percent control. PI3K activity was enhanced by up to 7-fold by IGFBP-3, which was significantly decreased by pretreatment with scavenger receptor-B1 neutralizing antibody (SRB1-Ab) (P<0.05, n = 3). **B**. Expression of the novel cell death receptor was significantly lower in HMVECs compared to the expression of scavenger receptor-B1 (SRB1) (P<0.0001, n = 3).

### IGFBP-3 Promotes Vasodilation that is Blocked by eNOS Inhibition

To examine the effects of IGFBP-3 on vasodilation, we tested the effects of the intraluminal application of IGFBP-3 on pressure induced constriction. In response to an intraluminal pressure of 70 mmHg, the vessels constricted and an application of IGFBP-3 resulted in a concentration-dependent decrease in myogenic constriction. This effect was significant at 100 (P<0.02, n = 5) and 300 ng/ml (P<0.01, n = 6) (Figure 3A), concentrations of free (unbound) IGFBP-3 likely to be seen in healthy humans. In subsequent experiments a concentration of 100 ng/ml was used to evaluate the effects of IGFBP-3 on myogenic tone with intraluminal pressures ranging from 10 to 100 mmHg. Myogenic constriction developed at pressures of 40, 70, and 100 mmHg and was significantly lower in the presence of intraluminal IGFBP-3 than vehicle (P<0.05 at all three different pressures, n = 6) (Figure 3B). Intraluminal application of 300 µM L-NAME increased the myogenic tone and blocked the effects of IGFBP-3 on myogenic tone (Figure 3B). Previously, we showed that IGFBP-3 directly activates the high density lipoprotein (HDL) receptor, scavenger receptor-B1 (SRB1) [28]. Thus, when SRB1-Ab was applied intraluminally (concentration 1∶100) with IGFBP-3, arterial tone was increased and IGFBP-3 did not affect myogenic tone (Figure 3B), indicating that the vasodilatory effects of IGFBP-3 are mediated through SRB1.

In addition to pressure, pharmacological constriction using agonists are key to evaluating vascular function. Rat PCAs were pressurized to 10 mmHg, to minimize the activation of myogenic mechanisms of constriction. Intraluminal application of IGFBP-3 (100 ng/ml) significantly attenuated serotonin-induced constriction (P<0.02 two-way ANOVA) (Figure 3C). In the presence of SRB1-Ab, IGFBP-3 did not reduce serotonin-induced constriction (Figure 3C).

**Figure 10:**
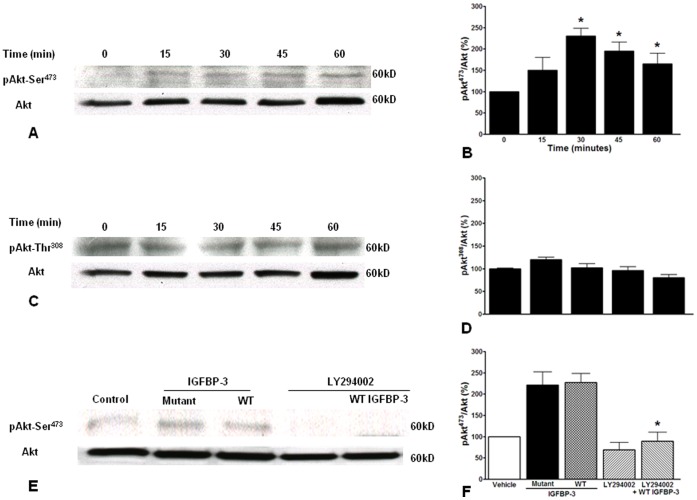
IGFBP-3 causes Akt phosphorylation at Ser^473^. **A**. Representative western blot depicting the time-course of Akt phosphorylation at Ser^473^ residue by 100 ng/ml IGFBP-3 and the bar graph (**B**) showing the pAkt^473^/Akt following IGFBP-3 stimulation (*P<0.01, n = 3). **C**. Representative western blot depicting the time-course of Akt phosphorylation at Thr^308^ residue by 100 ng/ml IGFBP-3 and the bar graph (**D**) showing the pAkt^308^/Akt following IGFBP-3 stimulation (n = 3). **E**. Representative western blot depicting the Akt-Ser^473^ phosphorylation by wild type (WT) and IGF-1 non-binding mutant of IGFBP-3 and the effects of PI3K inhibitor LY294002 (30 µM). **F**. WT and mutant IGFBP-3 phosphorylated Akt-Ser^473^ to similar extents and the phosphorylation by IGFBP-3 was blocked by LY294002 (*P<0.001, n = 3).

### IGFBP-3 Stimulates NO Release in Intact Arteries

When rat PCAs were loaded with DAF-FM and pressurized at an intraluminal pressure of 70 mmHg, intraluminal application of IGFBP-3 (100 ng/ml) dilated the arterial segments. This was accompanied by an increase in DAF-FM fluorescence (n = 5, Figure 4). In the presence of intraluminal 300 µM L-NAME, dilation in response to IGFBP-3 was not observed and no significant change was observed in DAF-FM fluorescence (P<0.03, Figure 4). The intraluminal presence of SRB1-Ab similarly blocked the effects of IGFBP-3 on DAF-FM fluorescence (P<0.02, Figure 4). While the SRB1-Ab blocked the effects of IGFBP-3, to our knowledge is has not been reported that SRB1is expressed in rat cerebral arteries. Thus, to confirm that SRB1 is expressed in the endothelium of rat cerebral arteries, real-time PCR was performed. Expression of rat SRB1 was detected in RNA obtained from intact arteries (CT: SRB1- 29.0±0.2; β-actin- 22.8±0.2, *n* = 3) (Figure 5A). However, because total RNA was obtained from intact arterial segments that include smooth muscle cells, we performed immunohistochemistry to distinguish the localization of this receptor from either the smooth muscle or endothelium. SRB1 immunofluorescence (red) was apparent in endothelial cells, which was identified by their horizontal alignment to the direction of blood flow and by immunofluorescence of eNOS (green) (Figure 5B). SRB1 was not observed in smooth muscle cells, identified by their perpendicular alignment to the direction of flow (Figure 5B), although, faint non-specific SRB1 immunofluorescence was observed in cell nuclei.

**Figure 11:**
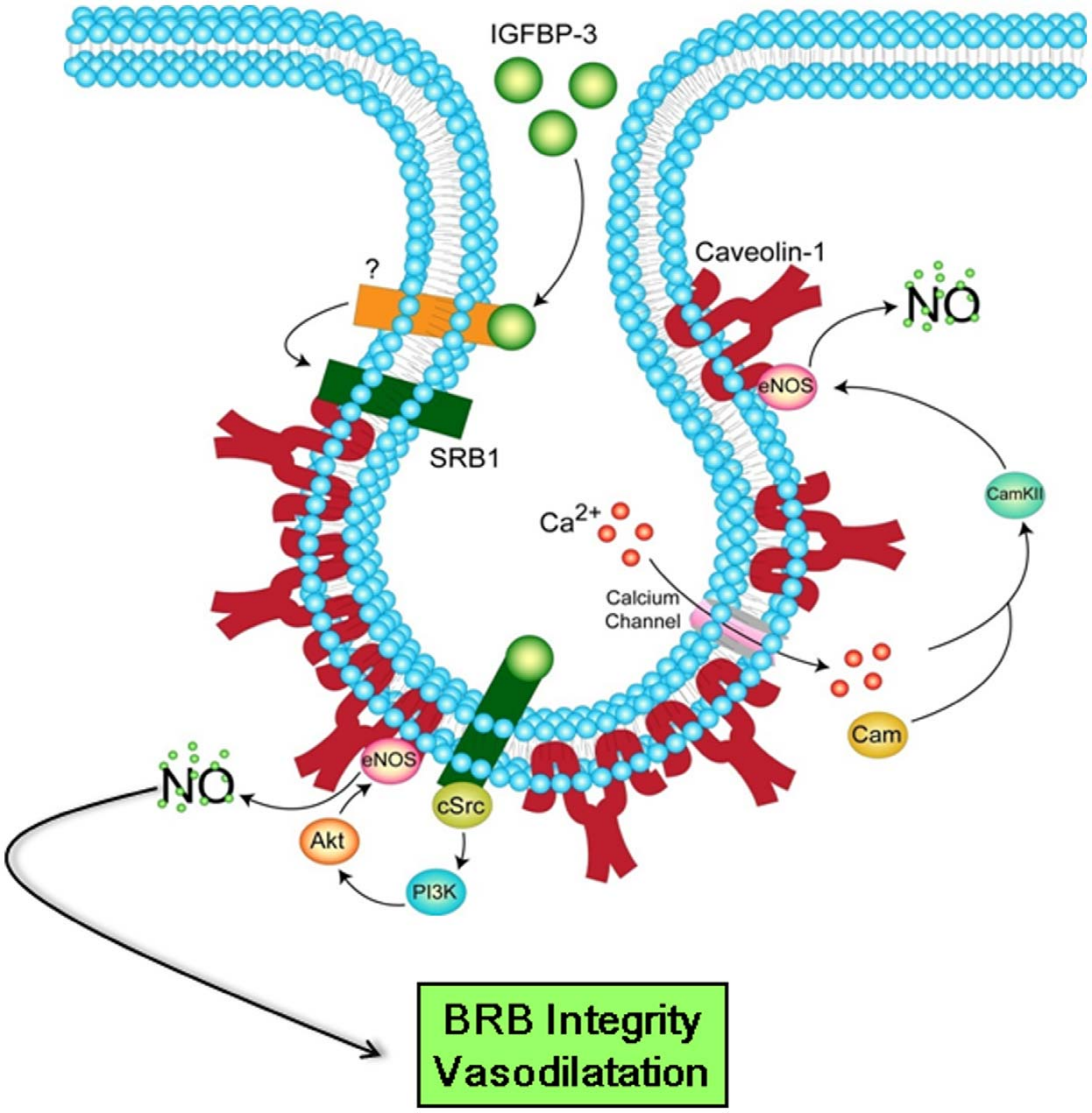
Schematic of cellular mechanisms of vasodilatation and protection of BRB integrity by IGFBP-3 via NO release. In endothelial cells, IGFBP-3 may directly activate the SRB1 or indirectly by releasing an unknown factor. Activation of SRB1 by IGFBP-3 activates eNOS via independent mechanisms. Ca^2+^-dependent pathway involves influx of Ca^2+^ via TRPV4 channels. Ca^2+^-calmodulin complex causes dephosporylation of Thr495 residue of eNOS resulting in the dissociation of the activated eNOS from caveolae however, this did not occur in microvascular endothelial cells. Ca^2+^-independent pathway involves activation of PI3K most likely by cSrc-kinase; PI3K activates Akt, which in turn activates eNOS by phosphorylation at Ser^1177^. NO released by IGFBP-3 mediates protective effect on BRB and vasodilatory functions by the PI3K/AKT pathway.

### Activation of eNOS and NO Release by IGFBP-3 are Independent of its Binding to IGF-1

IGFBP-3 is known to have IGF-1-independent effects. As shown above, IGFBP-3 increases NO generation and others have shown that IGF promotes NO release. To test whether eNOS activation and NO release by IGFBP-3 are dependent on its binding to IGF-1, we tested the effects of mutant IGFBP-3 (Figure 6A) that does not bind to IGF-1 [24]. In HMVECs, as expected wild type (WT) IGFBP-3 stimulated eNOS activity, expressed as the amount of conversion of [^14^C]L-arginine to [^14^C]L-citrulline that was inhibited by L-NAME. Mutant IGFBP-3 stimulated these responses to similar extents; this effect was significantly decreased by pretreatment with SRB1-Ab (Figure 6B). Stimulation with either WT or mutant IGFBP-3 resulted in an increase in DAF-FM fluorescence to a similar extent. Ionomycin, which activates eNOS by increasing calcium influx produced a robust increase in DAF-FM fluorescence as did both WT and mutant IGFBP-3. These responses were blocked by 300 µM L-NAME or SRB1-Ab (Figure 6C and D).

### NO Release by IGFBP-3 is Independent of Intracellular Calcium

However, it is not known whether intracellular calcium ([Ca^2+^]_i_) is involved in IGFBP-3- dependent eNOS activation and subsequent NO release. Fura-2 ratiometric determination of [Ca^2+^]_i_ was carried out by fluorescence microscopy in HMVECs. A robust increase in [Ca^2+^]_i_ was observed when HMVECs were stimulated with 10 µM 4αPDD, a selective activator of the nonselective cation channel TRPV4 [29] (Figure 7A and 7B). However, exposure to 100 ng/ml mutant IGFBP-3, a concentration that stimulated eNOS activity and NO release, did not increase [Ca^2+^]_i_ (Figure 7A and 7B). Western blotting studies revealed that IGFBP-3 treatment resulted in the dephosphorylation of eNOS at Thr^495^ and the effect was similar to that produced by 4αPDD (Figure 7C and 7D). Therefore, IGFBP-3 can activate eNOS by Ca^2+^-independent dephosphorylation of the Thr^495^ residue.

To further confirm that the Ca^2+^/CamKII pathway is not involved in NO release by IGFBP-3, the effect of KN93, a known inhibitor of CamK-II was evaluated on NO generation by 4αPDD and IGFBP-3. Treatment with 10 µM 4αPDD increased NO generations as assessed by DAF-FM fluorescence and this effect was inhibited by KN93, but not by KN92 the negative control of KN93 (Figure 8A and 8B); In contrast, NO generation by IGFBP-3 was not reduced by pretreatment with either KN93 or KN92 (Figure 8A and 8B).

### IGFBP-3 Activates PI3K/Akt Pathway Via SRB1

Previously, we observed that treatment with IGFBP-3 phosphorylated eNOS at Ser^1177^, causing its activation [28]. To delineate the signaling pathway involved in this response, we evaluated PI3K activity and phosphorylation of Akt following IGFBP-3 exposure. IGFBP-3 enhanced PI3K activity in HMVECs and this activity was inhibited by pretreatment with 1∶100 dilution of SRB1-Ab (P<0.05) (Figure 9A), supporting that SRB-1 mediates this effect. However, IGFBP-3-mediated actions can also occur via activation of a newly discovered cell death receptor, which while capable of activating initiator caspase-8 in cancer cells [26] can also mediate anti-inflammatory effects in healthy endothelial cells [30]. Real-time PCR revealed that the expression of this receptor was extremely low compared to that of SRB-1 in the endothelial cells used in our study (Figure 9B). Although, we cannot entirely exclude the involvement of this receptor, its effects should not have been blocked by SRB1 antibody, thus suggesting that the cell death receptor was not involved in the release of NO by IGFBP-3.

IGFBP-3 induced Akt phosphorylation on Ser^473^ with a peak response (2.5 fold increase) at 30 minutes that was maintained above basal levels for up to 60 minutes (Figure 9A and 9B); however, Akt phosphorylation on Thr^308^ was not significantly changed up to 60 minutes following the treatment with IGFBP-3 (Figure 10C and 10D). Both WT and IGFBP-3NB stimulated phosphorylation of Akt-Ser^473^ to similar extents and phosphorylation was blocked by pretreatment with the PI3K inhibitor, LY294002 (30 µM, P<0.001) (Figure 10E and 10F). Previously, we observed that treatment with IGFBP-3 phosphorylated Ser^1177^ on eNOS, causing its activation [28]. Our current study shows, for the first time, that this occurs via the PI3K/Akt pathway and is independent of IGF-1 binding.

## Discussion

In this study, we present four novel findings. First, as assessed by increased intraluminal HRP retention, expression of IGFBP-3 by retinal endothelial cells improves BRB barrier function. Second, IGFBP-3 protects endothelial tight junction protein complexes from VEGF-induced disruption. Third, IGFBP-3 independent of IGF-1 action, relaxes pressure and serotonin induced constrictions. Fourth, this IGF-1 independent vasodilatory response is independent of [Ca^2+^]_i_ but requires activation of SRB1 and PI3K as well as phosphorylation of Akt -Ser^473^. These novel actions are tightly linked to the ability of IGFBP-3 to stimulate physiological NO generation by the endothelium. A summary of these findings is illustrated in Figure 11.

NO has been implicated in the regulation of the BRB as the transporter for L-ariginine, the precursor of NO, is expressed at the inner BRB. One of the limitations of our study is that we did not directly test the effect of NO blockade on IGFBP-3 to enhance BRB function. However, we did examine the signaling pathways mediating its vasodilatory effects. In endothelial cells, a predominant pathway involved in agonist-induced eNOS activation involves increases in intracellular [Ca^2+^]_i_ for the activation of calmodulin. CamKII activates eNOS by dephosphorylating Thr^495^ residue [31]. Src-kinase-dependent activation of eNOS has also been shown to involve the CamKII pathway by increasing [Ca^2+^]_i_ via TRPV4 channels in endothelial cells as well as the PI3K/Akt pathway (Figure 11) [32]–[34]. However, our current studies support that IGFBP-3 does not stimulate NO generation by activating CamKII or increasing [Ca^2+^]_i_.

The beneficial effect of IGFBP-3 on the integrity of BRB is mediated by eNOS and not by iNOS. High levels of NO generated by iNOS disrupts BRB by proinflammatory effects and by down regulating the tight junction proteins, claudin and VE-cadherin [35], [36]. The vasodilatory and anti-inflammatory responses by low levels of NO produced by eNOS protect BRB and prevents disintegration of junctional protein complexes. This response is confirmed in the current study and this proposition is in agreement with our recent studies in two adult mouse models of retinal permeability [37]. However, we did not carry out these studies in the OIR model as the changes observed could be attributable to IGFBP-3 mediated developmental remodeling rather than the enhanced BRB integrity.

The current study evaluated the effects of IGFBP-3 on constriction mediated by intraluminal pressure and serotonin. Intraluminal pressure is a physiological stimulus that represents the basis of pressure-dependent autoregulation of organ blood flow and constitutes peripheral vascular resistance [18]. Cerebral arteries have been shown to be highly efficient in the pressure-dependent regulation of tone, which regulates vascular resistance and organ perfusion. IGFBP-3 attenuated both pressure- and agonist-induced constriction via SRB1-dependent endothelial NO release. NO-dependent vasodilation is a clear indicator that IGFBP-3 can enhance blood flow. We examined the effects of IGFBP-3 by intraluminal application because under normal physiological conditions IGFBP-3, circulates in the blood and bathes the entire endothelium. Thus, the effects we observed would be predictive of what occurs *in vivo,* and the doses of IGFBP-3 we used would be considered low and physiological, but certainly not pharmacological.

IGFBP-3 mediated actions are complex as IGFBP-3 has a variety of binding partners both on the cell surface and within cells, which are indispensible for its actions. The mid-region of IGFBP-3, which is the least conserved region among IGFBPs 1–6, is responsible for this cell surface binding. IGFBP-3 exerts its biological IGF/IGF-1R-independent actions through interaction with these binding partners [38], [39]. IGFBP-3 binds to the low-density lipoprotein receptor-related protein-1 (LRP-1)/α2M receptor, autocrine motility factor (AMF)/phosphoglucose isomerase (PGI) caveolin and transferrin/transferrin receptor [40]–[42]. The functional significance of these IGFBP-3 binding partners on the IGF/IGF-1R independent actions remains incompletely understood. However, they likely facilitate IGFBP-3 internalization and subsequent biological actions in both cytoplasmic and nuclear compartments. Moreover, IGFBP-3 has been shown to have diverse actions depending on the microenvironment, such as inhibition of cell growth and induction of apoptosis through interactions with nuclear proteins, including retinoid X receptor (RXR)-α, retinoic acid receptor (RAR), and Nur77 [43]. IGFBP-3-mediated apoptosis both in vitro and in vivo may occur via the activation of a novel cell death receptor that activates initiator caspase-8 [26]. As we show in the current study, our cells also express low levels of mRNA for this receptor; thus, we cannot exclude its involvement in our studies.

While our studies support the involvement of SRB1 in the vasodilatory effects of IGFBP-3, the possibilities remain that other receptors may be involved and activation of SRB1 by IGFBP-3 may be indirect through an unknown factor. Our studies ruled out IGF-1 as its binding was not required for the observed IGFBP-3 effects; however, IGFBP-3 is known to activate VEGF and IGF-1 release by endothelial cells [12], [44]. We believe that this is not likely to be the cause of NO release in the present study, as the effects of these growth factors are mediated by their specific receptor, and their activation should not have been blocked by SRB1-Ab. While not directly tested in our system, the possibility remains that IGFBP-3 binding to SRB-1 may be necessary for IGFBP-3 to activate VEGF and IGF-1 release, which then results in the NO release we observed. Interestingly, SRB1 has been shown to mediate the vascular effects of HDL via PI3K/Akt-dependent eNOS activation [32], [45] and Li et al [46] reported similar findings in CHO cells. SRB1 activation by HDL activates eNOS via SRB1 by increasing intracellular ceramide levels, whereas in HMVECs, eNOS activation was Akt-dependent and [Ca^2+^]_i_-independent. The current study shows that IGFBP-3 is a novel activator of SRB1 and that stimulation of eNOS occurs with low physiological concentrations of IGFBP-3. This response is independent of [Ca^2+^]_i_ and is consistent with what has previously been shown in endothelial cells by HDL-mediated activation of SRB1 [46]. Our studies further show that the signaling pathway downstream of the activation of SRB1 involves PI3K activation, which in turn phosphorylates Akt and that the Ser^473^ may mediate eNOS-Ser^1177^ phosphorylation and activation by IGFBP-3. Furthermore, we showed that NO generation via IGFBP-3 is independent of [Ca^2+^]_i_ and insensitive to the CamKII blocker. However, dephosphorylation of Thr^495^ was observed in endothelial cells treated with IGFBP-3, suggesting that the dephosphorylation occurred independent of the Ca^2+^/CamKII pathway. Activation of eNOS could also be achieved by the inhibition of PKC or tyrosine phosphatase, which have been shown to constitutively phosphorylate eNOS-Thr^495^; however this pathway was not explored further in the current study [47], [48].

Granata et al [12] previously showed that by stimulating IGF-1 release, IGFBP-3 at 10-fold higher concentrations than those used in this study activates SK activity and leads to the generation of S1P which has also been shown to increase NO generation. Previously, we showed that IGFBP-3 activates this sphingolipid system in both human CD34^+^ endothelial progenitor cells and HMVECs [28]. In CD34^+^ cells, IGFBP-3 exposure at a concentration of 100 ng/ml activated SK. This resulted in NO generation that was blocked by the selective SK inhibitor, D,L-threo-dihydrosphingosine [28]. We also showed that IGFBP-3 reduces apoptosis of endothelial cells and decreases production of proinflammatory factors [12], [28]. Collectively these studies suggest that the pathway mediating the vasoprotective effects of IGFBP-3 is likely both dependent on the particular concentration of IGFBP-3 used and the cell type tested.

While the liver contributes to serum IGFBP-3, IGFBP-3 is also expressed by both endothelial cells [49] and endothelial progenitor cells [19]. Following vascular injury IGFBP-3 release by the injured vessel stimulates recruitment of endothelial progenitor cells from bone marrow into the circulation to support vessel repair. Thus IGFBP-3 likely has both autocrine and paracrine effects. Our current study shows a direct effect of IGFBP-3 on the vascular wall suggesting that IGFBP-3 can have direct vasoprotective effects largely due to the promotion of NO generation. Thus, IGFBP-3 appears to be an efficient hypoxia-regulated physiological stimulus for angiogenic and vasoreparative processes. Interestingly, the expression of SRB1 is increased by erythropoietin, a hypoxia-regulated factor released by ischemic tissue and serves to facilitate the local effect of IGFBP-3 to both produce NO and re-establish blood flow.

The local release of IGFBP-3 following injury may represent a generalized compensatory mechanism or a response to cellular or tissue stress that is readily adaptable to diverse and adverse stimuli. Furthermore, the effects of IGFBP-3 are clearly concentration-dependent. At high concentrations, for example, as have been observed in cancer microenvironments, IGFBP-3 release can serve a beneficial role by inducing apoptosis of cancer cells, restoring tissue homeostasis. Moreover, not only are tissue levels of IGFBP-3 critical but higher circulating IGFBP-3 levels were shown to confer protection from cancer [50] but recently this was brought into question [51].

Moreover, the diverse set of IGFBP-3 binding partners also supports the pleotrophic effects of this factor. Recently, humanin, a 24 amino acid peptide that inhibits neuronal cell death was identified as an IGFBP-3 binding partner [52]. While our studies support the vasoprotective effects of IGFBP-3 to be mediated by SRB-1, a role for the other IGFBP-3 receptors in the vasculature cannot be entirely excluded [26], [53].

In summary, the current study shows that IGFBP-3 over expression by the retinal endothelium restores BRB integrity following hyperoxia-induced injury and corrects the retinal morphology of OIR mice towards normal. When applied intraluminally, IGFBP-3 independent of IGF-1, has a concentration-dependent effect on reducing vasoconstriction developed in response to either intraluminal pressure or vasoconstrictive agonists via the stimulation of NO release via SRB1 activation. IGFBP-3 activates eNOS by both Ca^2+^-independent dephosphorylation of Thr^495^ residue and phosphorylation of Ser^1177^ residue via the PI3K/Akt pathway. This study suggests that IGFBP-3 directly affects vascular tone and that the levels of IGFBP-3 present in the sera of healthy individuals may represent a physiological mechanism to sustain vascular health.
